# 蔬菜水果中植物化学物质防治肺癌作用及机制研究现状

**DOI:** 10.3779/j.issn.1009-3419.2017.12.08

**Published:** 2017-12-20

**Authors:** 甜甜 郭, 聪敏 刘, 肇妤 高, 宇彤 贺

**Affiliations:** 050000 石家庄，河北医科大学第四医院肿瘤研究所 Cancer Institute, the Fourth Hospital of Hebei Medical University/The Tumor Hospital of Hebei Province, Shijiazhuang 050000, China

**Keywords:** 肺肿瘤, 蔬菜水果, 植物化学物质, 抗肿瘤, Lung neoplasms, Vegetables and fruits, Phytochemicals, Antineoplastic

## Abstract

无论是在全球还是我国，肺癌都是严重危害人类健康的恶性肿瘤。研究表明肺癌与环境因素以及生活方式密切相关，流行病学发现多吃蔬菜水果可以预防肺癌。蔬菜水果中含有丰富的植物化学物质，如异硫氰酸酯类、吲哚类、黄酮类等。这些植物化学物质通过调节与抗肿瘤相关的通路从而抑制肿瘤细胞增殖、诱导肿瘤细胞凋亡，降低肺癌发生的危险性。本文旨在对蔬菜水果中的植物化学物质在肺癌发生发展中的作用机制进行综述，为更好地预防和治疗肺癌提供理论依据与方向。

## Abstract (en)

Whether in the world or China, lung cancer is a malignant tumor which is harmful to human health. There were studies showed that lung cancer is tightly related to the environment factors and life style. The epidemiology study found that eating more fruits and vegetables can prevent lung cancer. Vegetables and fruits are rich in phytochemicals such as isothiocyanates, indoles, flavonoids and so on. These phytochemicals reduce the risk of lung cancer by modulating antitumor-related pathways such as inhibition of cell proliferation, induction of apoptosis, and the like. The aim of this review is to summarize the mechanisms of phytochemicals in vegetables and fruits in the pathogenesis and progression of lung cancer, so as to provide theoretical basis and direction for the prevention and treatment of lung cancer.

肺癌是目前全球发病率与死亡率均居第一位的恶性肿瘤。据GLOBOCAN 2012估计，全球肺癌新发病例为182万，占全部恶性肿瘤的12.9%；死亡病例159万，占全部恶性肿瘤的19.4%^[1]^。在我国，肺癌也是发病率和死亡率最高的恶性肿瘤，2015年肺癌发病例数达73万，死亡例数达61万^[2]^。

研究表明肺癌与环境因素以及生活方式密切相关。Tu等^[3]^和Vieira等^[4]^运用流行病学的方法研究了蔬菜水果与肺癌发生风险之间的关系。其中Tu等^[3]^进行了一项涉及到2, 139例非小细胞肺癌（non-small cell lung cancer, NSCLC）病例和2, 163例在食物食用频率与肺癌病例匹配的对照人群，研究结果显示蔬菜水果可以降低32%的肺癌风险比值比（odds ratio, OR），为0.68，95%CI为0.55-0.85。Vieira等^[4]^也进行了一项蔬菜水果与肺癌发生风险相关的*meta*分析，蔬菜水果高剂量与低剂量进行比较得出蔬菜水果总相对危险度（relative risk, RR）值为0.86（95%CI: 0.78-0.94），蔬菜的RR为0.92（95%CI: 0.87-0.97），水果的RR值为0.82（95%CI: 0.76-0.89）。Li等^[5]^还研究了蔬菜水果在中国男性肺癌患者生存预后中的意义。结果显示虽然新鲜蔬菜水果的摄入与肺癌死亡风险之间没有统计学意义，但是在NSCLC、小细胞肺癌亚组中的分析结果显示，与小细胞肺癌患者相比，NSCLC患者食用蔬菜水果的频率较低（OR=0.62, 95%CI: 0.41-0.95）。有研究^[7, 8]^提示蔬菜水果降低肺癌发生风险可能的机制是蔬菜水果中含有丰富的植物化学物质，例如异硫氰酸酯类、吲哚类、黄酮类等，植物化学物质可以通过不同的机制调节与抗肿瘤相关的通路从而抑制肿瘤细胞增殖、诱导肿瘤细胞凋亡，降低肺癌发生的危险性。本文旨在综述蔬菜水果中植物化学物质在肺癌发生发展中的作用机制，从而更好地为预防和治疗肺癌提供方向。

## 1 异硫氰酸酯类

异硫氰酸酯（isothiocyanate, ITC）是由硫代葡萄糖苷经黑芥子酶水解形成的天然小分子，广泛存在于十字花科蔬菜中。大量研究表明异硫氰酸酯可以通过诱导肿瘤细胞周期阻滞、促进肿瘤细胞凋亡；诱导内质网应激和细胞自噬；诱导活性氧的产生等多种机制抑制肿瘤的生长、侵袭和转移^[9]^。

Mori等^[10]^为了研究异硫氰酸酯与肺癌发生风险之间的关系，进行了一项前瞻性调查研究。该研究主要对82, 330名（男性：38, 663名，女性：43, 667名）年龄在45岁-74岁的参与者进行研究，对所有参与者每日摄入的138种食物的食用频率进行问卷调查，其中包括6种十字花科蔬菜以及3种腌制的十字花科蔬菜。经过14.9年的随访后，共有1, 499名（男性1, 087名，女性412名）参与者发生了肺癌。数据分析结果显示十字花科蔬菜摄入与肺癌风险之间存在显著负相关，分层分析发现从未吸烟者风险比（hazard ratio, HR）为0.49（95%CI: 0.27-0.87, *P*=0.04）；过去吸烟者的HR为0.59（95%CI: 0.35-0.99, *P*=0.10）。

萝卜硫素（SFN）是ITCs的一种，近年来，有很多的研究者致力于探索其在肺癌发生发展中的作用。早期研究表明，SFN通过抑制1期代谢酶和诱导2期代谢酶阻断肿瘤的起始阶段，影响肿瘤发生和发展^[11, 12]^。随后，Zhou等^[13]^用SFN处理宣威肺腺癌细胞株（XWLC-05）细胞，观察到G_0_期/G_1_期细胞的百分比下降，G_2_期/M期细胞的百分比增加，细胞凋亡百分比达到27.6%。Liang等^[14]^和Zuryn等^[15]^也对SFN抑制细胞增殖，诱导细胞凋亡方面进行了研究，其中Zuryn等^[15]^使用不同浓度的SFN处理NSCLCA549细胞株后，同样可观察到G_2_期/M期细胞数量增加。Jiang等^[16]^利用SFN处理两种不同的肺癌细胞A549和H1299 48 h，结果显示SFN不仅可以增加G_0_期/G_1_期和G_2_期/M期的细胞数量，使细胞停滞在S期，诱导细胞凋亡，SFN还能抑制组蛋白脱乙酰酶（histone deacetylase inhibitor, HDAC）的活性，提高肺癌细胞乙酰化组蛋白H3和H4的水平，从而减缓肺癌细胞的生长速度。

近年来，研究者们从分子机制方面对SFN的抗肿瘤作用进行了探讨。Zhu等^[17]^用不同浓度的SFN处理人肺癌细胞系A549和H1299 7 d后，发现SFN通过抑制miR-19的水平从而使Wnt/β-连环蛋白途径的负调节因子GSK3β的水平升高，继而降低Wnt/β-连环蛋白途径的活性，而Wnt/β-连环蛋白途径对于保持肿瘤干细胞（cancer stem cells, CSCs）的活性是至关重要的，所以SFN最终可抑制肺CSCs的活性。Li等^[18]^的研究同样表明SFN可抑制CSCs的活性，该研究用SFN处理H460细胞，通过比较对照组和SFN处理组的miRNA表达谱，可观察到经SFN处理的H460细胞中miR-214、miR-145、miR-199a和miR-199b显著上调，进一步的研究结果显示SFN是通过上调miR-214从而作用于c-MYC的编码区，继而抑制NSCLC的CSCs发挥作用。另外Wang等^[19]^同样用不同浓度的SFN处理具有高转移潜力的95D和H1299细胞株，可观察到这两种细胞的迁移能力减弱。通过尾静脉将95D和H1299细胞注射到裸鼠中，与对照组相比，可观察到经SFN处理的小鼠肺转移发生率明显降低。Wang等^[19]^研究进一步证明SFN可以通过组蛋白修饰降低miR-616-5p的表达水平，继而使得GSK3β/β连环蛋白通路失活，此外该过程还抑制了上皮间质转化（epithelial-mesenchymal transition, EMT）过程。

因此，以SFN为例的异硫氰酸酯类主要通过抑制细胞的增殖，诱导细胞凋亡，影响HDAC的水平从而抑制肺癌细胞的生长。在分子机制方面，SFN主要通过抑制miR-19继而降低Wnt/β-连环蛋白的活性，或通过上调miR-214从而作用于c-MYC的编码区来抑制NSCLC的CSCs的活性；SFN还可以通过降低miR-616-5p的表达水平，使得GSK3β/β连环蛋白通路失活抑制肺癌转移。

## 2 吲哚类

吲哚-3-甲醇（indole-3-carbinol, I3C）是十字花科蔬菜如白菜、甘蓝中一种主要的植物化学物质，且具有防癌和抗癌作用。早期的研究表明I3C在肺癌发生发展中的作用主要是抑制细胞的增殖以及诱导细胞凋亡。例如Kassie等^[20]^为了观察I3C对4-（甲基亚硝基氨基）-1-（3-吡啶基）-1-丁酮（NNK）加苯并（a）芘（BaP）诱导的A/J小鼠肺肿瘤的抑制作用，通过管饲给予小鼠NNK加BaP（每个2 μmol）的混合物每周2次，可引起每只小鼠发生（21.1±5.2）个肺癌病灶。在给予致癌物阶段同时给予小鼠含有不同浓度I3C的饮食，可观察到小鼠的肺癌病灶数目随着I3C浓度的增加而减少，最高可达75%，进一步分析结果显示，I3C可以降低由NNK+BaP所诱导的Akt活化水平以及其下游靶标BAD的磷酸化表达水平，这是一种细胞质信号转导蛋白，通过抑制凋亡从而发挥重要作用。此外I3C还可以降低增殖相关核抗原Ki-67以及PCNA的表达，该研究表明I3C对肺肿瘤的抑制作用部分是通过抑制细胞增殖和诱导凋亡来实现的。2010年Choi等^[21]^用I3C处理肺癌A549细胞株后同样也观察到I3C显著抑制了细胞的增殖以及诱导细胞的凋亡，进一步的研究结果显示，I3C通过增加P53以及细胞周期蛋白依赖激酶抑制剂（cylin dependent kinases inhibitors, CDKIs）的表达水平而使细胞停滞在G_0_期/G_1_期，并通过Fas以及半胱天冬酶-8级联通路的激活诱导细胞凋亡。这也是I3C对A549肺癌细胞凋亡作用分子机制的首次报道。

近几年，研究者们从放疗敏感性和化学预防效果等方面对I3C在肺癌发生中的作用进行了探讨。Xiao等^[22]^用I3C预处理EGFR表达阳性的人肺腺癌H1975和人肺鳞癌H226细胞，结果显示I3C明显降低了上述两种肺癌细胞对γ-射线照射的放射敏感性，还增加了两种细胞中EGFR蛋白的表达水平和Y845位点的磷酸化水平，而I3C对EGFR表达阴性的人肺鳞癌NIH-H520细胞的放射敏感性的影响非常小。在Qian等^[23]^的研究中，他们利用烟草致癌物诱导的A/J小鼠肺癌模型来评估I3C对肺腺癌的化学预防效果，结果表明，I3C是通过作用于受体酪氨酸激酶/PI3K/Akt信号通路来发挥作用的。但是在Dagne等^[24]^的研究中指出，虽然I3C在A/J小鼠中有降低肺癌发生的作用，但是这种作用随着体重的增加反而会减小，所以他们进一步研究了低剂量I3C与水飞蓟素的结合物是否同样存在着抑制肺癌的作用，结果表明I3C与水飞蓟素的结合物可以降低肺癌的发生，而两者单独作用时效果并不显著，另外两者的混合物还降低了细胞增殖相关蛋白ERK和Akt的表达水平，表明I3C与水飞蓟素可以通过调节这些蛋白质的水平从而降低肺癌的发生。Song等^[25]^和Qian等^[26]^从炎症诱发肺癌的角度进一步研究了I3C与水飞蓟素结合物所发挥的作用，其中Song等用烟草中的炎症因子4-甲基亚硝胺-1-（3-吡啶基）-1-丁酮（NMK）与脂多糖（lipopolysaccharides, LPS）处理的小鼠发生了（14.7±4.1）个肺癌病灶，而用NNK+LPS处理并给予I3C和水飞蓟素组合的小鼠的肺癌病灶数目为（7.1±4.5）个，降低了52%。此外，还观察到I3C与水飞蓟素结合物在降低肿瘤数目方面的作用也大于单独使用I3C。进一步研究表明I3C与水飞蓟素结合物的抗增殖作用也高于单个化合物，这些作用是通过靶向细胞周期蛋白D1、CDK2、CDK4和CDK6以及pRB实现的。提示我们I3C与水飞蓟素结合物对吸烟者肺癌的化学预防作用具有较重大的意义。

总之，I3C可以通过影响细胞增殖、细胞凋亡过程中的相关物质来抑制肺癌细胞的生长；增加EGFR表达阳性肺癌细胞中EGFR蛋白表达水平降低肺癌细胞的放射敏感性；通过作用于受体酪氨酸激酶/PI3K/Akt信号通路发挥化学预防作用。

## 3 黄酮类

研究^[27]^表明，经常摄入富含黄酮类化合物的蔬菜和水果可以降低肺癌、大肠癌和乳腺癌等肿瘤的发生率。木樨草素是一类天然的黄酮类化合物，广泛存在于蔬菜水果中，木樨草素降低肺癌危险性的机制主要包括抑制细胞增殖、细胞转移、促进细胞凋亡等方面。Chian等^[28]^的研究表明，裸鼠口服木樨草素后明显抑制了裸鼠中皮下生长的NSCLC细胞株A549细胞的异种移植瘤的生长。该研究进一步表明木樨草素不仅可以在体外抑制Nrf2通路，而且还可以在体内抑制Nrf2通路，从而抑制肿瘤细胞的增殖。Tan等^[29]^的研究是利用香烟烟雾提取物诱导正常人支气管上皮细胞，进而评估木樨草素对细胞的保护作用。结果显示木樨草素不仅可以降低氧化物的水平，同样可以抑制Nrf2通路。Chen^[30]^和Ruan等^[31]^的研究均表明木樨草素可以作用于EMT，抑制E-钙粘蛋白的下降，从而降低癌细胞的迁移能力。Choi等^[32]^用白介素4（interleukin-4, IL-4）处理鼠巨噬细胞细胞株RAW 264.7，而后用不同浓度的木樨草素处理细胞，可观察到木樨草素以一种剂量依赖的形式表现出对IL-4所诱导的STAT6的磷酸化的抑制作用，继而可以抑制M2样肿瘤相关巨噬细胞（tumor-associated macrophage, TAM）表型。木樨草素还可抑制由IL-4刺激所增强的趋化因子（C-C基序）配体2（CCL2）的分泌。结果表明木樨草素可通过抑制TAM分泌的IL-4增强的CCL2发挥出抑制单核细胞募集以及肺癌细胞的迁移作用。Lee等^[33]^从TAM受体酪氨酸激酶（receptor tyrosine kinase, RTK）Tyro3、Axl和MerTK方面展开研究，A549和H460细胞以及顺铂耐药A549/CisR和H460/CisR细胞经木樨草素处理后可观察到木樨草素以剂量依赖的方式降低A549和A549/CisR细胞中TAM RTK的三种蛋白质水平，同样的，H460和H460/CisR细胞中，木樨草素处理后Ax1和Tyro3的蛋白水平也降低。该研究表明TAM RTKs是木樨草素抑制细胞增殖的治疗靶点，并降低了NSCLC细胞的化学耐药性。关于木樨草素促进肿瘤细胞凋亡的研究，Park^[34]^和Lee等^[35]^的细胞学研究表明，木樨草素可触发内质网应激相关的凋亡和细胞非正常自噬，从而诱导细胞凋亡。Cho等^[36]^也研究了木樨草素在诱导肺癌细胞凋亡中的机制，从改善放射治疗效果角度出发，用木樨草素处理NCI-H460和NCI-H1299两种NSCLC细胞株，之后对细胞进行γ-电离辐射（infrared radiation, IR）的照射。结果显示在促进NSCLC细胞死亡方面，木樨草素/IR的组合比单独的使用木樨草素或IR处理更有效。木樨草素和IR联合治疗可以促进细胞的凋亡，该过程与Bcl-2的下调和半胱天冬酶-3、-8和-9的活化有关，此外两者的联合作用还可以抑制p38 MAPK通路从而降低ROS的产生，无论是抑制p38 MAPK还是减少ROS的含量均可以减少细胞的凋亡和半胱天冬酶-8和-9的活化。另外在裸鼠NCI-H460细胞异种移植瘤模型中，与对照组相比，木樨草素/IR实验组的肿瘤生长延迟了21.8 d，细胞的凋亡同样增加。总之该研究结果显示木樨草素通过激活p38/ROS/半胱天冬酶级联来诱导细胞凋亡，从而可以作为放射增敏剂。

Ma等^[37]^用不同浓度梯度的木樨草素处理NCI-H460细胞，结果显示木樨草素以浓度依赖的方式诱导细胞的凋亡，调节蛋白Bax/Bcl-2的表达水平，抑制凋亡相关蛋白Bad的表达。另外Sirt1的表达水平经木樨草素处理后同样降低，而Sirt1调节着NCI-H460细胞的凋亡。Meng等^[38]^的研究同样表明木樨草素可以抑制肺癌细胞增殖，诱导细胞的凋亡，此过程伴随着半胱天冬酶-3和-9的活化增加、Bcl-2的降低、Bax表达的升高以及MEK及其下游激酶ERK的磷酸化以及Akt的激活。进一步研究表明木樨草素通过激活MEK-ERK信号通路，显著抑制肺癌细胞的迁移。除了上述研究外，还有一些研究者探索了木樨草素在致癌物诱导的模型中所发挥的作用。Kasala等^[39]^在动物水平上评估了木樨草素对苯并（a）芘诱导的肺癌发生的抗氧化和抗肿瘤潜力。苯并（a）芘处理的小鼠体内的脂质过氧化物升高，抗氧化物水平下降，而经过木樨草素处理后，这些抗氧化物的表达水平显著提高，并维持细胞的正常状态。此外，木樨草素还能有效地抵消苯并（a）芘诱导的PCNA，细胞色素P450 1A1（recombinant cytochrome P450 1A1, CYP1A1）和核因子κB（nuclear factor kappa B, NF-κB）的升高。以上这些发现提示木樨草素对苯并（a）芘诱导的肺癌具有化学预防的潜力。此外，木樨草素在六价铬[Cr（Ⅵ）]所诱导的肺癌发病中也发挥着作用，木樨草素通过清除ROS以及调节与ROS相关的多种细胞信号传导通路保护肺部细胞免受铬的损害^[40]^。

综上，以木樨草素为例的黄酮类，主要通过抑制Nrf2通路抑制肺癌的生长，通过抑制EMT、CCL2激活MEK-ERK通路等降低肺癌的迁移能力，可触发内质网应激相关的凋亡和细胞非正常自噬，影响凋亡相关蛋白水平诱导细胞凋亡，降低TAM RTK蛋白水平以降低化学耐药性，通过激活p38/ROS半胱天冬酶级联来诱导细胞凋亡，从而可以作为放射增敏剂。

本文主要综述了蔬菜水果中主要植物化学物质在降低肺癌发生风险方面的研究，这些植物化学物质不仅可以抑制肺癌细胞的增殖、侵袭和转移，诱导细胞的凋亡，还可以改善放射治疗效果，降低化学耐药性，在一定程度上起到化学预防的作用，虽然这些研究目前还都停留在细胞以及动物水平上，但这也为今后这些有益的植物化学物质应用于临床提供了方向，应引起学者的广泛关注并进行深入研究，为预防治疗肺癌提供理论依据。
